# Identification of subjects with polycystic ovary syndrome using electronic health records

**DOI:** 10.1186/s12958-015-0115-z

**Published:** 2015-10-29

**Authors:** Victor Castro, Yuanyuan Shen, Sheng Yu, Sean Finan, Cindy Ta Pau, Vivian Gainer, Candace C. Keefe, Guergana Savova, Shawn N. Murphy, Tianxi Cai, Corrine K. Welt

**Affiliations:** Information Systems, Massachusetts General Hospital, Boston, MA USA; Biostatistics, Harvard School of Public Health, Boston, MA USA; Department of Medicine, Brigham and Women’s Hospital, Boston, MA USA; Informatics, Children’s Hospital Boston, Boston, MA USA; Reproductive Endocrine Unit, Massachusetts General Hospital, Boston, MA 02114 USA; Neurology, Massachusetts General Hospital, Boston, MA USA; University of Utah, Division of Endocrinology, Metabolism and Diabetes, EIHG 15 N 2030 E, Salt Lake City, 84112 USA

**Keywords:** Natural language processing, ICD9 code, Hyperandrogenism, Polycystic ovary morphology

## Abstract

**Background:**

Polycystic ovary syndrome (PCOS) is a heterogeneous disorder because of the variable criteria used for diagnosis. Therefore, International Classification of Diseases 9 (ICD-9) codes may not accurately capture the diagnostic criteria necessary for large scale PCOS identification. We hypothesized that use of electronic medical records text and data would more specifically capture PCOS subjects.

**Methods:**

**S**ubjects with PCOS were identified in the Partners Healthcare Research Patients Data Registry by searching for the term “polycystic ovary syndrome” using natural language processing (*n* = 24,930). A training subset of 199 identified charts was reviewed and categorized based on likelihood of a true Rotterdam PCOS diagnosis, i.e. two out of three of the following: irregular menstrual cycles, hyperandrogenism and/or polycystic ovary morphology. Data from the history, physical exam, laboratory and radiology results were codified and extracted from notes of definite PCOS subjects. Thirty-two terms were used to build an algorithm for identifying definite PCOS cases and applied to the rest of the dataset. The positive predictive value cutoff was set at 76.8 % to maximize the number of subjects available for study. A true positive predictive value for the algorithm was calculated after review of 100 charts from subjects identified as definite PCOS cases with at least two documented Rotterdam criteria. The positive predictive value was compared to that calculated using 200 charts identified using the ICD-9 code for PCOS (256.4; *n* = 13,670). In addition, a cohort of previously recruited PCOS subjects was submitted for algorithm validation.

**Results:**

Chart review demonstrated that 64 % were confirmed as definitely PCOS using the algorithm, with a 9 % false positive rate. 66 % of subjects identified by ICD-9 code for PCOS could be confirmed as definitely PCOS, with an 8.5 % false positive rate. There was no significant difference in the positive predictive values using the two methods (*p* = 0.2). However, the number of charts that had insufficient confirmatory data was lower using the algorithm (5 % vs 11 %; *p* < 0.04). Of 477 subjects with PCOS recruited and examined individually and present in the database as patients, 451 were found within the algorithm dataset.

**Conclusions:**

Extraction of text parameters along with codified data improves the confidence in PCOS patient cohorts identified using the electronic medical record. However, the positive predictive value was not significantly different when using ICD-9 codes or the specific algorithm. Further studies are needed to determine the positive predictive value of the two methods in additional electronic medical record datasets.

**Electronic supplementary material:**

The online version of this article (doi:10.1186/s12958-015-0115-z) contains supplementary material, which is available to authorized users.

## Background

Polycystic ovary syndrome (PCOS) is the most common endocrine disorder in reproductive age women. The diagnosis is based on its cardinal features, including irregular menstrual cycles, hyperandrogenism and polycystic ovary morphology, with two out of three features required for the diagnosis in the absence of other disorders causing the same symptoms [1, 2]. Additional features are variable, with obesity exacerbating hyperandrogenism and risk for metabolic disorders including impaired glucose tolerance, type 2 diabetes and metabolic syndrome [3–5]. The associated features depend on the diagnostic criteria employed [5–7], which differ depending on the specialty of the recruiting physician [8].

Based on the heterogeneous features and the need to rule out other diagnoses before PCOS is ascertained, it may be difficult to use codified data from the electronic medical record to confidently identify patients with PCOS. The most readily available identifier, the International Classification of Diseases, 9^th^ edition code (ICD-9), misclassified 13–20 % of adolescents with PCOS [9]. Conversely, PCOS was confirmed as a diagnosis in only 73 % of adolescents in a separate study [10]. In adult women identified with PCOS using an ICD-9 code, 28 % had documented anovulation and clinical hyperandrogenism in the record, whereas an additional 52 % had only one of these features documented [11]. Additional validation is needed to determine whether the ICD-9 code is accurate in identifying adult women with PCOS.

Other codified data that could be useful to corroborate the diagnosis of PCOS include laboratory measurements and ultrasound results. However, an elevated androgen level is not necessary for a diagnosis of PCOS in the setting of clinical hyperandrogenism and measured levels may be altered by treatment. Further, while laboratory tests could be used to exclude patients with other diagnoses, the results may not be electronically available. Current procedural technology codes may be available to indicate that a pelvic ultrasound was performed. However, the necessary ultrasound parameters for the diagnosis of polycystic ovary morphology, such as ovarian volume, are not typically codified data and cannot be captured as confirmatory information for a PCOS diagnosis. Taken together, the specificity of the ICD-9 code when at least one available confirmatory PCOS feature was available in the electronic medical record is approximately 70–80 %, but other confirmatory codified data may not be readily available. Therefore, more extensive analyses may be needed to identify women with PCOS in electronic medical records.

Natural language processing takes electronic free text and codifies the data into computationally functional categories [12]. These categories can be used to establish diagnostic features useful for selecting women with PCOS and confirming the presenting features. We identified a cohort of women with PCOS using ICD-9 codes and identified a second cohort using natural language processing along with codified data. The primary outcome of the study was a comparison of the positive predictive value of the PCOS diagnosis using the ICD-9 code compared to an algorithm used to identify PCOS that incorporated natural language processing and codified data. The secondary outcome was validation of the algorithm cohort using a previously identified, well-phenotyped cohort of subjects with PCOS. The data highlight the utility and limitations of using natural language processing to accurately identify large sets of women with PCOS.

## Methods

### Data source

The primary data source was the Partners Healthcare Research Patients Data Registry (RPDR), spanning more than 20 years of data from 4.2 million patients. The database contains over 227 million encounters, 193 million coded ICD-9 diagnoses, 105 million medications, 200 million procedures, 852 million lab values and over 55 million unstructured clinical notes, which are a combination of outpatient visit notes, inpatient discharge summaries, radiology reports, and others. The RPDR population is approximately 55 % female, 72 % Caucasian and patients have an average age of 45.7 with a standard deviation of 23.2 years.

We initially identified women with PCOS using the ICD-9 code 256.4 in the RPDR database (*n* = 13,670). Two hundred randomly identified charts were reviewed individually for confirmation of a PCOS diagnosis. Twelve records had no notes, labs or ultrasounds available, and were not included in the final count.

Subsequently, an initial, broadly defined dataset (referred to as the broad data ‘mart’; *n* = 265,481) was identified using the ICD-9 code for PCOS and other potentially relevant ICD-9 codes for inclusion and exclusion of polycystic ovary syndrome (Additional file 1: Figure S1 and Table S1). Women, aged 18 to 74 years, with more than one longitudinal medical record note greater than 50 characters were included in the search. Inclusion codes were PCOS (256.4), menstrual disorders (626.x), female infertility (628.x), hirsutism (704.1), alopecia (704.00), acne (706.x) and diabetes complicating pregnancy (648.0x) (Additional file 1: Table S1 and Figure S1). Ovarian procedures, including wedge resection (65.22 and 65.24), medications (topical acne agents, metformin and isotretinoin) and laboratory tests (high testosterone and DHEAS) were also included.

A second refined datamart was created, which included women between the ages of 18 and 40 years with at least one mention of the term ‘PCOS’ in a clinical note at Massachusetts General Hospital (MGH) or Brigham and Women’s Hospital (BWH) (refined datamart; Additional file 1: Figure S1). Women with a history of fibroids (ICD-9 654.1*), ovarian cysts (ICD-9 620.2*), any eating disorder (307.1*, 307.5*), premature ovarian failure (ICD-9 256.3*), Cushing syndrome (ICD-9 255.0*), endometriosis (ICD-9 617.*) or a history of elevated prolactin (LOINC group: PRL), 17 hydroxy progesterone (LOINC group: 17OHPROG), urine free cortisol (LOINC group: U-F) or follicle-stimulating hormone (LOINC group: FSH) were excluded from the refined datamart (Additional file 1: Figure S1 and Table S2).

The data marts consisted of all electronic records for study patients stored using the i2b2 software (i2b2 v1.6.04; USA) [13]. The i2b2 system is a scalable computational framework for managing human health data and the Workbench facilitates analysis and visualization of such data. The Partners Institutional Review Board approved all aspects of this study and the usual safeguards for human subjects’ data were applied.

### Training Set

The full electronic medical record of 50 women sampled randomly from the initial broadly defined datamart and 199 women sampled randomly from the refined datamart population were reviewed by a board-certified clinician investigator (CKW). Patients were classified as definite PCOS, probable PCOS, definite NOT PCOS, or not enough information. A subset of 20 notes from the refined datamart was reviewed by an additional investigator to assess inter-rater reliability of the sample (CCK). For patients classified as true cases (definite or probable), related signs and symptoms, comorbidities and other phenotypes were abstracted from the medical record to inform feature selection and model training for NLP analysis (Table 1).

**Table 1:** Polycystic ovary syndrome related signs, symptoms, comorbidities, medication, laboratory results, ultrasound findings and other phenotypes abstracted from the medical record to inform feature selection and model training for natural language processing (NLP) analysis

Feature	Parameter	Source
PCO morphology	Ovarian volume >10	Pelvic ultrasound
PCO morphology	≥12 follicles or PCO morphology in text	Pelvic ultrasound
Hyperandrogenism	Elevated testosterone, DHEAS or androstenedione	Laboratory data
Hyperandrogenism	Hirsutism	Note
Hyperandrogenism	Ferriman Gallwey Score	Physical exam
Hyperandrogenism	Acne	Physical exam or note
Hyperandrogenism	Alopecia, Hair loss, balding	Physical exam or note
Irregular menses	Cycle length	Note
Irregular menses	Irregular menses, oligomenorrhea, amenorrhea, etc.	Note
Hyperandrogenism	Clitoromegaly	Physical exam
Associated Features		
Acanthosis Nigricans	Acanthosis	Physical exam
Gestational Diabetes	Gestational diabetes	Note
Infertility	Anovulatory infertility	Note
Obesity	Obesity	Physical exam or note
Type 2 diabetes	Type 2 diabetes	Laboratory data or note
Pertinent Negatives		
Excessive exercise	Exercise history	Note
Chronic opioid or drug use	Substance history	Note
Hypothalamic amenorrhea	BMI or Hypothalamic amenorrhea history	Physical exam or note

### NLP analysis

An expert-defined list of terms (custom dictionary) was created including clinically-relevant phenotypic features of PCOS (i.e. ‘alopecia’, ‘hirsutism’), terms related to comorbidities of PCOS (‘obesity’, ‘infertility’) as well as terms related to potential competing diagnosis (i.e. ‘Cushing’s syndrome’, ’eating disorder’, hypothalamic amenorrhea). The terms were then mapped to the Systemized Nomenclature of Medicine-Clinical Terms (SNOMED-CT), a hierarchically organized clinical health care terminology index with over 300,000 concepts, to allow for variations in language use, or the RxNorm, a normalized naming systemic for generic and branded drugs.

Outpatient notes, discharge summaries, radiology reports, operative notes and pathology reports were then processed using the clinical Text Analysis and Knowledge Extraction System (cTAKES) [14], which processes clinical text notes and identifies a term mentioned in the text, along with qualifying attributes (i.e., negated, non-negated, current, history of, family history of). We computed the number of times each term was mentioned across all notes for each patient.

### Training a classification algorithm

A proportional odds kernel machine (POKM) regression procedure for ordinal outcomes prediction [15] was performed on the training set of 198 subjects with available data. The training set consisted of 46 features and the chart-reviewed gold standard PCOS label taking three ordinal levels: definite PCOS (PCOS_D_), probable PCOS (PCOS_P_) and no PCOS. The POKM with Gaussian kernel, incorporating non-linear effects of the predictors, improves the prediction performance of the final classification algorithm. The tuning parameters required in the modeling were selected based on the cross-validation and Akaike information criteria as discussed previously [15]. The algorithm was applied to the remaining subjects in the refined datamart and probabilities of having PCOS_D_ and PCOS_P_ were assigned to each subject. A subject is classified as PCOS positive if the predicted probability of having PCOS_D_, ${p}_{\mathrm{PCO}{\mathrm{S}}_D}$, exceeds a threshold value. The threshold value was chosen to ensure that among those classified as PCOS positive, 75 % have PCOS_D_.

### Controls

Patients with at least one visit to a women’s health clinic at MGH or BWH and no mention of the term PCOS in a clinical note and no history of clinically-relevant features of PCOS were selected as controls for the study (control pool). Patients selected by the classification algorithm were then matched 1:10 to women in the control group on the basis of age, gender, number of recorded events (diagnosis, procedures lab tests and medications) and earliest and most recent visit in the health system.

### Validation

For PCOS subjects identified using ICD-9 codes and predicted definite PCOS and probable PCOS using the algorithm, 200 and 191 charts were reviewed, respectively. The number of chart-review validated PCOS subjects was determined to provide a true positive predictive value. A diagnosis of PCOS was confirmed if at least two of the following three features were present: 1) history or physical exam evidence of hirsutism, acne or alopecia, or an elevated total testosterone or DHEAS level [5], 2) irregular menses as documented in the history, and/or 3) polycystic ovary morphology on ultrasound reports consisting of a volume of at least 10 mL in an ovary without a dominant follicle or cyst and/or a description of a large number of follicles [1]. Presence of one confirmatory feature was considered probable PCOS. Presence of an exclusionary diagnosis, such as anorexia nervosa, hypothalamic amenorrhea or primary ovarian insufficiency was considered definitely not PCOS.

In addition, a list of medical records from subjects recruited with PCOS for a previous study (*n* = 693) was submitted to determine whether they appeared in the PCOS subject dataset after the algorithm was applied [16]. These subjects had physical exam, laboratory and ultrasound data that confirmed a diagnosis of PCOS by the NIH criteria, as previously described [5].

## Results

Using ICD-9 codes, 200 charts (total *n* = 13,670) were examined to identify confirmatory criteria for the diagnosis of PCOS (Table 2). A total of 132 subjects had 2 confirmatory findings that documented the diagnosis of PCOS, while 29 had one confirmatory finding. The positive predictive value was 74 % for definite PCOS and 90 % for definite and probable PCOS. Of those with two confirmatory findings, 84 % had PCOS documented using NIH criteria. Twenty-two subjects had no confirmatory information for the diagnosis of PCOS. There was a 9.5 % false positive rate, with exclusionary diagnoses including primary ovarian insufficiency (*n* = 4), endometriosis (*n* = 1), hyperprolactinemia (*n* = 2), premenstrual dysphoric disorder (*n* = 1), eating disorder (*n* = 3), hypothalamic amenorrhea (*n* = 1) opioid use (*n* = 1), pituitary tumor (*n* = 1), mistaken diagnosis (*n* = 2) or family history, only (*n* = 1).

**Table 2:** Comparison of true polycystic ovary syndrome (PCOS) on chart review in women with PCOS determined using ICD-9 codes or using an algorithm incorporating natural language processing and codified data

Method	ICD-9 Code	PCOS Algorithm-definite	PCOS algorithm-probable	*P* value*
Number of Charts	200	150	41	
Chart Reviewed Definite PCOS (%)	132 (66)	98 (65)	25 (61)	0.2*
Chart Reviewed Probable PCOS (%)	29 (14.5)	33 (22)	7 (17)	0.2
Not PCOS (%)	17 (8.5)	10 (7)	8 (20)	0.9
Unable to Determine (%)	22 (11)	9 (6)	1 (2)	0.04

For the algorithm PCOS diagnoses, Definite or Probable were defined by probability cutoff levels. Chart reviewed definite PCOS had at least two confirmatory diagnostic criteria to support the diagnosis and probable had at least one confirmatory criterion

*The *p* value for the algorithm was calculated using both the definite and probable categories

In contrast, an initial review of random notes from the broad datamart (Additional file 1: Table S1) identified only 1/17 (5.8 %) with a confirmed diagnosis of PCOS. Differences included a broad range of diagnostic codes used to widen the pool for subsequent algorithm development and no upper age limit. Therefore, age less than 45 at the time of diagnosis or presenting feature was added as an inclusion criteria and eating disorders were added to the exclusion criteria (*n* = 178,510). However, only 6/50 (12 %) subjects had definite or probable PCOS. The proportion of definitely positive PCOS subjects was too low to proceed with algorithm development, based on previous experience [17].

In the refined datamart, a total of 13,077 patients met the criteria for the study population after exclusions were applied (Additional file 1: Table S2). The refined datamart overlapped with the broad datamart (71 %), but included additional subjects not identified using codified data (Additional file 1: Figure S1). Of the 200 randomly-selected patients in the training set, 93 (46.5 %) were classified as definite PCOS and 59 (29.5 %) as probable PCOS. There were 17 subjects who did not have PCOS for an 8.5 % false positive rate. Thirty-one subjects (15.5 %) did not have available information to confirm the diagnosis of PCOS. The positive predictive value was 85 %, for definite and probable PCOS, similar to that using the ICD-9 codes (*p* = 0.7).

### Algorithm results

The data from 198 subjects in the training set were evaluated with the cTAKES results. Data were collapsed into 36 NLP and 14 codified terms after removing terms that were not found in at least 10 % of subjects. Using these terms, the area under the curve of the algorithm for classifying PCOS_D_ was 0.87. A cutoff of 0.392 was chosen to achieve a positive predictive value of 0.75 for PCOS_D_ and to maximize the number of subjects identified. The positive predictive value for definite/probable PCOS was 91 % (95 % confidence intervals: 0.84-0.96). This cut-off value classifies 6295 patients in the data mart (48.6 %) as definite PCOS.

A subset of 150 charts from subjects with definite PCOS and 41 charts from subjects with probable PCOS were reviewed based on previous studies in diseases with a similar prevalence (Table 2) [17, 18]. When the definite and probable PCOS categories were combined, the review demonstrated a positive predictive value of 96 %. Further, stringent requirements for documentation of two Rotterdam criteria in the record resulted in a 68 % positive predictive value. The majority of these definite PCOS subjects (81 %) also met the NIH criteria for PCOS. The false positive rate was 10 %. The validated categories were not different using ICD-9 codes, extracting subjects using the term “PCOS” in the electronic medical record or using the algorithm (*p* = 0.2). However, the proportion of subjects for which the diagnosis of PCOS could not be determined was significantly lower (5 vs 11 %; *p* < 0.04).

### Validation results

Of the 693 subjects with PCOS recruited through a previous study, 451 were present in the broad datamart and a subset of 201 was present in the refined datamart. The majority of subjects with PCOS recruited for the previous study did not appear in the datamart because they did not have a sufficient number of notes; they were not patients in the Partner’s system (*n* = 178), were seen at a Partners hospital other than MGH or BWH (*n* = 12), or were employees (*n* = 26). The second most common reason for non-inclusion in the datamart was a documented mildly elevated prolactin that was subsequently normal (*n* = 17), was drawn during pregnancy or postpartum (*n* = 3) or was drawn after starting medication that raised prolactin after participation in the study (*n* = 3). Two subjects had an elevated urine free cortisol but were confirmed not to have Cushing syndrome. None of the PCOS subjects appeared in the control set.

### Demographics of cases in the validated cohort and controls

Cases were slightly younger than controls (Table 3). The cases were also less likely to have had a pregnancy documented at one of the Partner’s hospitals. The difference may be based on the design of the study, because all controls were required to have presented for a visit for women’s health. The lifetime maximum BMI was also greater in the cases than in the controls.

**Table 3:** Demographics of subjects chosen for the refined datamart

		PCOS Cases			PCOS Controls		*p* value
	N	6,295			59,456		
		Proportion			Proportion		
Gender	Female	1.00			1.00		
Age	18-25	0.15			0.11		
	26-35	0.45			0.37		
	36-45	0.35			0.33		
	46-55	0.05			0.13		
	56-65	0.00			0.06		0.03
Insurance	Private	0.71			0.67		
	Public-Medicaid	0.05			0.08		
	Public-Medicare	0.01			0.02		
	Public-Other	0.08			0.11		
	Other	0.09			0.10		
	Unknown	0.05			0.03		0.9
Race	White	0.63			0.64		
	Asian	0.07			0.06		
	Black	0.08			0.08		
	Hispanic	0.11			0.10		
	Other	0.11			0.11		1.0
Pregnancy	(Partners Hospital System)	0.36			0.56		0.007
Pap Smear	(lifetime history)	0.29			0.21		0.3
Smoker	(ever smoked)	0.08			0.05		0.6
Type 2 Diabetes	(lifetime history)	0.08			0.02		0.1
Hypertension	(lifetime history)	0.10			0.09		1.0
Womens' Health Visit	(lifetime history)	0.72			1.00		<0.001
		Mean	SD		Mean	SD	
Age	current	33.55	7.23		36.93	9.75	<0.001
BMI	lifetime max	30.99	9.02		26.85	6.49	<0.001
		Median	Q25	Q75	Median	Q25	Q75
Observation Period Start	year, median (IQR)	2003	1998	2008	2004	1999	2008
Observation Period End	year, median (IQR)	2012	2010	2013	2011	2010	2012
Number of facts	count, median (IQR)	186	68	428	226	90	524

Controls were slightly older, with a lower BMI. Other factors were not different

## Discussion

Using IDC-9 codes or identifying subjects using an algorithm consisting of terms identified in electronic medical records along with codified data from definite PCOS subjects resulted in no significant difference in the positive predictive value for identification of PCOS subjects. However, the use of the algorithm resulted in fewer subjects with absent documentation confirming the PCOS diagnosis. Thus, the use of ICD-9 codes or an algorithm incorporating terms pertinent to the PCOS diagnosis results in a reasonable rate of identifying true cases with PCOS in the Partners Healthcare RPDR. Nevertheless, the use of the developed algorithm may improve confidence in large scale collections and data inquiry by removing indeterminate subjects in studies of PCOS.

There has been no systematic evaluation of the rate of true PCOS subjects identified using ICD-9 codes in adults. Our data suggest that the positive predictive value for PCOS is better than that from previous findings in adolescents with PCOS [9]. In contrast to a 13–20 % misclassification rate, only 8.5 % of subjects in the Partners Healthcare RPDR database were misclassified based on ICD-9 codes, although an additional 11 % did not have confirmatory data in the notes to make a clear PCOS status determination. These data suggest that ICD-9 codes may provide a reasonable proxy for true PCOS subjects if validated in other health record systems.

In contrast to the use of the ICD-9 code for PCOS, ICD-9 codes that identified features of PCOS such as irregular menses, hirsutism and acne were too broad to identify women with documented PCOS. Instead, using the term “polycystic ovary syndrome” in the electronic medical record in the refined datamart included subjects not defined by the ICD-9 code but with a greater specificity for PCOS, similar to ICD-9 coding alone. Taken together, the current electronic medical records database suggests that using broad catchment coding diagnoses would not be specific enough to capture subjects with true PCOS from an electronic medical records cohort.

Despite the moderately accurate performance of the ICD-9 code, greater confidence may be needed when using the collected datasets for analysis of fertility, associated medical problems and PCOS features or for collection of anonymized blood samples for study of genetics. The use of an algorithm containing codified data and parameters identified using the clinical Text Analysis and Knowledge Extraction System has the potential to greatly improve the confidence in patient identification. Previous studies demonstrate the superiority of cTAKES/codified data compared to ICD-9 codes for identifying subjects for large scale studies, with ROC characteristics increasing from 54 to 87 % in studies of depression [18]. Similarly, for inflammatory bowel disease, ROC characteristics improved from 86–89 % to 95–96 % [19]. Remarkably, there was a 38 % improvement in identification of rheumatoid arthritis patients using the cTAKES/codified data algorithms. We did not demonstrate such a remarkable improvement in PCOS patient identification using the same method. However, confirmation relied on physician documentation of the cardinal features of PCOS and these were not available in many charts. If the algorithm had been set at a higher cutoff for the positive predictive value, the proportion of definite PCOS subjects would have been greater, but at the expense of subject number.

The advantages of identifying PCOS subjects using cTAKES along with codified data are many. There can be very poor understanding of PCOS among physicians, and misclassification or missed diagnoses are common [20]. As an example in the current study, an ICD-9 code for PCOS was used during a work up that ultimately revealed an exclusionary diagnosis. On the other hand, there can be failure to understand that an elevated laboratory testosterone level is not necessary to make a diagnosis [21] and the ICD-9 code may not be used to indicate a diagnosis that is truly PCOS. These types of patients may be captured by words documented in electronic medical records during the work up. Previous studies also demonstrate that the specialty of the provider influences the criteria required to make a diagnosis of PCOS [8], resulting in missed diagnoses in some cases. The ability to analyze text may override some of these problems depending on the completeness of the notes utilized.

Indeed, the completeness and detail of the available electronic medical records are the most important factors limiting the algorithm method for identification of PCOS subjects. The algorithm relies on terminology, physical exam findings and an appropriate work up for PCOS. The use of templates that ensure proper documentation may not be flexible if they are not set up for the diagnosis of PCOS. As endorsed by an evidence-based methodology workshop on PCOS [2], providers are encouraged to use the Rotterdam criteria and to document the criteria through which the diagnosis of PCOS was made. If adopted, these measures will increase the power of the identification algorithm for large scale recruitment and data analysis. In addition, documenting menstrual cycle parameters [22] will also increase the ability to detect PCOS patients.

## Conclusions

Within the Partners Healthcare RPDR, an algorithm developed using cTAKES and codified data compared with ICD-9 codes resulted in similar positive predictive values for identifying patients with PCOS. However, the algorithm improved confidence in PCOS case identification. The algorithm will be validated in an independent health care system to evaluate the performance with different health care providers and documentation. If validated, the algorithm may prove an invaluable tool for confident accrual of large numbers of women with PCOS.

## Additional file

Additional file 1: Figure S1. Datamart calibration. The circles represent A) the initial broad datamart identified using codified data, B) the second refined datamart in which electronic notes with the words polycystic ovary syndrome or PCOS were found, and C) patients from the entire Research Population Data Registry database, without codified exclusion criteria. The overlap represents patients that were found using both codified data and with a PCOS term in the note (AXB) or patients with a PCOS term in the note and without exclusion criteria (BXC). Of note, patients without exclusion criteria are also found in A and AXB, but are not shown here for clarity. The numbers in the orange circles represent the number of charts with a confirmed PCOS diagnosis over the total number of charts reviewed by an expert (CKW) and the percentage confirmed. The white box indicates the patients with evaluable charts who were not included in the broad definition datamart (no codified terms identified) but who did have a PCOS term in their note and were included in the refined datamart. **Table S1.** ICD 9 codes for diagnoses and procedures and laboratory values used for inclusion and exclusion in the broad PCOS datamart. Patients were all female, 18-74 years of age (current), with any of the listed parameters measured at Massachusetts General Hospital or Brigham and Women’s Hospital. **Table S2.** Inclusion and exclusion criteria used to create the second refined PCOS datamart. Patients were all female, 18-40 years of age at first identification of any listed parameter from records at Massachusetts General Hospital or Brigham and Women’s Hospital. (DOCX 36 kb)
