# Supplementary material for: Identification of subjects with polycystic ovary syndrome using electronic health records
Source: Reprod Biol Endocrinol. 2015 Oct 29;13:116. doi: 10.1186/s12958-015-0115-z (PMC4625743; doi:10.1186/s12958-015-0115-z)
Supplement: Additional file 1: Figure S1. — Datamart calibration. The circles represent A) the initial broad datamart identified using codified data, B) the second refined datamart in which electronic notes with the words polycystic ovary syndrome or PCOS were found, and C) patients from the entire Research Population Data Registry database, without codified exclusion criteria. The overlap represents patients that were found using both codified data and with a PCOS term in the note (AXB) or patients with a PCOS term in the note and without exclusion criteria (BXC). Of note, patients without exclusion criteria are also found in A and AXB, but are not shown here for clarity. The numbers in the orange circles represent the number of charts with a confirmed PCOS diagnosis over the total number of charts reviewed by an expert (CKW) and the percentage confirmed. The white box indicates the patients with evaluable charts who were not included in the broad definition datamart (no codified terms identified) but who did have a PCOS term in their note and were included in the refined datamart. Table S1. ICD 9 codes for diagnoses and procedures and laboratory values used for inclusion and exclusion in the broad PCOS datamart. Patients were all female, 18-74 years of age (current), with any of the listed parameters measured at Massachusetts General Hospital or Brigham and Women’s Hospital. Table S2. Inclusion and exclusion criteria used to create the second refined PCOS datamart. Patients were all female, 18-40 years of age at first identification of any listed parameter from records at Massachusetts General Hospital or Brigham and Women’s Hospital. (DOCX 36 kb) [file 12958_2015_115_MOESM1_ESM.docx]

Supplementary Figure 1. Datamart calibration. The circles represent A) the initial broad datamart identified using codified data, B) the second refined datamart in which electronic notes with the words polycystic ovary syndrome or PCOS were found, and C) patients from the entire Research Population Data Registry database, without codified exclusion criteria. The overlap represents patients that were found using both codified data and with a PCOS term in the note (AXB) or patients with a PCOS term in the note and without exclusion criteria (BXC). Of note, patients without exclusion criteria are also found in A and AXB, but are not shown here for clarity. The numbers in the orange circles represent the number of charts with a confirmed PCOS diagnosis over the total number of charts reviewed by an expert (CKW) and the percentage confirmed. The white box indicates the patients with evaluable charts who were not included in the broad definition datamart (no codified terms identified) but who did have a PCOS term in their note and were included in the refined datamart.

**(B)
Refined Datamart: PCOS term in note**

**N=24,930**

**(A)
Broad Datamart: Broad definition of PCOS**

**N=265,481**

**A∩B**

**N=17,820**

**7,110 with PCOS in note but not in datamart (9/38; 24%)**

**(C)
Patients without exclusion criteria**

**N>1 X10^6^**

**(B∩C)
N=13,077**

Supplementary Table 1. ICD 9 codes for diagnoses and procedures and laboratory values used for inclusion and exclusion in the broad PCOS datamart. Patients were all female, 18-74 years of age (current), with any of the listed parameters measured at Massachusetts General Hospital or Brigham and Women’s Hospital.

| **Parameter - Inclusion** | **Codified Data** |  |
| --- | --- | --- |
| **Diagnosis** | **Billing Code** | **Problem List Terms Associated with ICD 9 Code** |
| Polycystic ovary syndrome | 256.4 | Polycystic ovaries, Polycystic ovarian syndrome |
| Menstrual Disorders | 626.x | Menstrual disorder, Amenorrhea, Irregular menses, Irregular menstrual bleeding, Irregular uterine bleeding, Dysfunctional uterine bleeding, Irregular vaginal bleeding, Intermenstrual bleeding; Menorrhagia; Menometrorrhagia; Oligomenorrhea; Secondary amenorrhea |
| Female infertility | 628.0, 628.1, 628.8 | Infertility, Female infertility |
| Hirsutism | 704.1 | Hirsutism |
| Alopecia | 704.00 | Alopecia, Hair loss |
| Acne | 706.0, 706.1 | Acne vulgaris, Cystic acne, Acne |
| Diabetes mellitus complicating pregnancy, childbirth or the puerperium | 648.0x |  |
| **Diagnosis** | **Billing Code** |  |
| Ovarian procedures | 65.22, 65.24; CPT^1^: 58920, 58679, 58662 |  |
| Pelvic ultrasound | CPT-4:76856, 76857 | USPEL1; USPEL2; USPEL4^2^ |
| **Laboratory Tests** | **LOINC Group**^3^ | **Flag** |
| Testosterone | TES, TEST | HIGH |
| DHEA Sulfate | DHEAS | HIGH |
| **Medication** | **Sources** |  |
| Topical acne agents | ^4^ LMR/Oncall Outpatient Prescribing / Inpatient Pharmacy |  |
| Metformin | LMR/Oncall Outpatient Prescribing / Inpatient Pharmacy |  |
| Isotretinoin | LMR/Oncall Outpatient Prescribing / Inpatient Pharmacy |  |
| **Exclusion Parameter** |  |  |
| **Diagnosis** | **Billing Code** | **Problem List Terms Associated with ICD 9 Code** |
| Fibroids | 654.1x | Fibroids, Uterine fibroids |
| Ovarian cysts | 620.2 | Ovarian cyst |
| Early menopause/premature ovarian failure | 256.3 | Premature ovarian failure, Premature menopause |
| Cushing Syndrome | 255.0 | Cushing syndrome, Cushing's syndrome |
| Endometriosis | 617.x | Endometriosis |
| **Exclusion-Laboratory Tests** | **LOINC Group**^3^ | **Flag** |
| Prolactin | PRL | HIGH |
| 17 hydroxyprogesterone | 17 OH progesterone | >1000 ng/dL |
| Urine Free Cortisol | UFC | >100 mcg/dL |
| Follicle Stimulating Hormone | FSH | >20 IU/L |

^1^ CPT – current procedural terminology codes as published by the American Medical Association

^2^ USPEL –ultrasound of the pelvis

^3^ LOINC – logical observation identifiers names and codes for laboratory test orders and results

^4^ LMR – longitudinal medical record and Oncall – electronic medical records available at Massachusetts General Hospital and Brigham and Women’s Hospital

Supplementary Table 2. Inclusion and exclusion criteria used to create the second refined PCOS datamart. Patients were all female, 18-40 years of age at first identification of any listed parameter from records at Massachusetts General Hospital or Brigham and Women’s Hospital.

| **Inclusion Criteria** |  |  |
| --- | --- | --- |
| Female Gender |  |  |
| Living (October 2012) |  |  |
| At least one clinical document at Massachusetts General Hospital or Brigham and Women’s Hospital^1^ |  |  |
| **Note Criteria** | **Note Universe** | **Search Terms** |
| Mention of PCOS in a clinical note | Any non-weight center note^2^ in which patient is 18-40 years old at the time of the note | PCOS, Poly[ ]cystic ovar* |
| **Exclusion Criteria** |  |  |
| **Diagnosis** | **Billing Code** | **Problem List Terms Associated with ICD 9 Code** |
| Fibroids | 654.1x | Fibroids, Uterine fibroids |
| Ovarian cysts | 620.2 | Ovarian cyst |
| Early menopause/premature ovarian failure | 256.3 | Premature ovarian failure, Premature menopause |
| Cushing Syndrome | 255.0 | Cushing syndrome, Cushing's syndrome |
| Endometriosis | 617.x | Endometriosis |
| Eating disorders | 307.1x, 307.5x | Eating disorder, Bulimia, Anorexia Nervosa |
| **Exclusion-Laboratory Tests** | **LOINC Group**^3^ | **Flag** |
| Prolactin | PRL | HIGH |
| 17 hydroxyprogesterone | 17 OH progesterone | >1000 ng/dL |
| Urine Free Cortisol | UFC | >100 mcg/dL |
| Follicle Stimulating Hormone | FSH | >20 IU/L |

^1^ Hospital sites covered by the IRB approved study

^2^ Weight center notes employed a review of systems template that incorporated the terminology polycystic ovary syndrome. Review of 20 notes demonstrated that the coding resulted in false positive results for PCOS. Therefore, weight center notes were removed from the note universe.

^3^ LOINC – logical observation identifiers names and codes for laboratory test orders and results
